# Retrospective Evaluation of Patients Admitted to the Emergency Department Due to Anaphylaxis in Children: A Single-Center Study from Türkiye

**DOI:** 10.3390/children13020203

**Published:** 2026-01-31

**Authors:** Emre Aygün, Ezgi Yalçın Güngören, İrem Çırpıcı, Sevgi Sipahi Çimen

**Affiliations:** 1 Department of Pediatrics, Şişli Hamidiye Etfal Training and Research Hospital, University of Health Sciences, 34371 Istanbul, Turkey; irem.cirpici@saglik.gov.tr; 2 Department of Pediatric Allergy/Immunology, Şişli Hamidiye Etfal Training and Research Hospital, University of Health Sciences, 34371 Istanbul, Turkey; ezgi.yalcin4@saglik.gov.tr (E.Y.G.); sevgi.sipahicimen@saglik.gov.tr (S.S.Ç.)

**Keywords:** anaphylaxis, children, emergency department, epinephrine, quality indicators

## Abstract

**Highlights:**

**What are the main findings?**

- Pediatric anaphylaxis management in the emergency department shows sub-stantial gaps in timely epinephrine administration and discharge planning.
- Prescription rates of epinephrine auto-injectors and referrals to allergy specialists remain suboptimal despite guideline recommendations.

**What is the implication of the main finding?**

- Targeted educational and system-level interventions are needed to improve adherence to anaphylaxis management guidelines in pediatric emergency settings.
- Standardized discharge protocols may reduce preventable morbidity and re-current anaphylaxis in children.

**Abstract:**

Background: Management of pediatric anaphylaxis in the emergency department remains clinically important. The research investigated pediatric anaphylaxis medical indicators together with physician adherence to international treatment protocols at a Turkish tertiary medical center. Methods: Between September 2014 and July 2025, 166 pediatric anaphylaxis patients were retrospectively reviewed for triggering factors, clinical findings, treatment approaches, and quality indicators. Results: The mean age of the patients was 7.4 ± 5.6 years. Food allergy was the main cause with 53%, followed by drugs with 24.7%. Food allergy in infants was 85.7%, while drug reactions in adolescents reached 37.2% (*p* < 0.001). Skin findings were present in 93.4% of the patients, and respiratory symptoms were present in 67.5% of the patients. Epinephrine was administered to 97.6% of patients, 95.2% of whom were given intramuscularly. The rate of epinephrine administration in the first 30 min was 61.1%. Drug-induced anaphylaxis showed the highest proportion of severe cases (81.6%, *p* < 0.001). A biphasic reaction was seen in 6%. The auto-injector prescription rate was 7.8%, and the allergist referral rate was 15.7%. No deaths were observed. Conclusions: While acute-phase management largely adheres to international guidelines, significant gaps persist in post-discharge care with low auto-injector prescription and allergist referral rates.

## 1. Introduction

Anaphylaxis is a rapidly developing and potentially life-threatening systemic hypersensitivity reaction [1]. Epidemiological studies indicate that the incidence of anaphylaxis in children has increased substantially in recent years, reaching 50–112 cases per 100,000 person-years in population-based analyses [2]. The lifetime prevalence ranges from 0.3% to 5.1%. Notably, these rates are increasing in the pediatric age group [3]. Foods are the most common triggers of anaphylaxis in childhood—particularly cow’s milk, eggs, and nuts—whereas the proportion of drug-induced reactions increases with age [1,4]. Additionally, idiopathic anaphylaxis, where the triggering factor cannot be identified, is observed in approximately 10–20% of children [3].

The pathophysiology of anaphylaxis involves the rapid release of vasoactive mediators, such as histamine, from mast cells and basophils. This process may be initiated through IgE-mediated immunological mechanisms or non-IgE-mediated pathways, including activation of the MRGPRX2 receptor [5,6]. Clinical manifestations commonly involve the skin, respiratory, gastrointestinal, and cardiovascular systems; in severe cases, hypotension, bronchospasm, and shock may occur [7]. Immediate intramuscular administration of epinephrine is the cornerstone of treatment and is strongly recommended by international guidelines, including those from EAACI, WAO, and AAAAI [7,8]. Despite adrenaline being such a critical drug, the picture is not very encouraging when we look at its use in pediatric emergency departments. In most clinics, the rate hovers around 30–50 per cent. However, current data from tertiary centres show much higher figures, ranging from 94.8 per cent to 100 per cent [3]. Notably, drug-induced anaphylaxis is associated with higher mortality rates than other trigger types, with antibiotics accounting for approximately half of fatal reactions [4,9].

Despite well-established guideline recommendations, substantial variability persists between centers in both acute management and post-discharge preventive practices, including epinephrine auto-injector prescription, patient and caregiver education, and referral to allergy specialists. Emergency settings in Türkiye now have enough data to identify patterns of pediatric anaphylaxis according to recent research. Caliskan et al. found that medications stood as the leading cause of allergic reactions, which totalled 54% of all cases while cow’s milk allergies occurred most frequently in infants at 33.3% of food-related cases and doctors prescribed auto-injectors to 75.3% of patients when they left the hospital [3]. However, comprehensive assessments of emergency department quality indicators across multiple domains remain limited in the Turkish pediatric population. Although pediatric anaphylaxis epidemiology and emergency management strategies have been reported in various countries, data from Türkiye remain limited, particularly regarding quality indicators such as timing and dosing of epinephrine administration, observation duration, and post-discharge care in emergency department settings [7].

Previous research has established the epidemiological patterns of pediatric anaphylaxis but there is a lack of systematic assessments that evaluate quality indicators, including epinephrine administration timing and dosage, observation period length, auto-injector distribution rates, and specialist referral patterns. The research fills this knowledge gap through its complete assessment of pediatric emergency department clinical practices and international quality standards compliance in a Turkish tertiary hospital.

Therefore, this study aimed to retrospectively evaluate the clinical characteristics, triggering factors, emergency management practices, and post-discharge outcomes of pediatric anaphylaxis cases presenting to a tertiary pediatric emergency department. By assessing adherence to international treatment standards and key quality indicators, this study seeks to identify critical gaps and potential areas for improvement in the management of pediatric anaphylaxis.

## 2. Materials and Methods

### 2.1. Study Population and Sample

This retrospective and descriptive single-center study included patients admitted due to anaphylaxis in the Pediatric Emergency Department of SBU Şişli Hamidiye Etfal Training and Research Hospital between September 2014 and July 2025. The sample size was not precalculated; all cases that met the inclusion criteria in the specified period were included in the study. Inclusion conditions were to be followed up in the emergency department with a pre-diagnosis or diagnosis of anaphylaxis, to be between the ages of 0 and 18, and to meet the diagnostic criteria for anaphylaxis according to EAACI 2021, Turkish National Anaphylaxis Guide 2018 or WAO 2020 criteria; these guidelines state that in addition to more than one system involvement, single-system involvement but severe respiratory or cardiovascular symptoms should also be considered as anaphylaxis [10,11,12]. It was required that there was sufficient information about diagnosis, treatment and observation in the file records. Exclusion criteria included non-anaphylaxis allergic reactions (e.g., urticaria alone or isolated angioedema), inadequate records, and cases over 18 years of age.

Age groups were classified as 0–2 years, 3–5 years, 6–11 years and 12–18 years. Triggering factors were grouped under the headings of food, drug, venom, aeroallergen and other/unknown. Food subgroups (cow’s milk, eggs, nuts, etc.) and drug subclasses (such as penicillin/cephalosporin, NSAIDs) were also specified from the file records. The severity of anaphylaxis was assessed as mild, moderate, or severe. Concomitant diseases—asthma, atopic dermatitis, allergic rhinitis, and others—were recorded.

When defining aeroallergen-induced anaphylaxis, we based our criteria on the following: systemic anaphylactic reactions occurring during documented exposure to airborne allergens (such as pollen, mould spores, animal dander) or within 30 min of such exposure. Of course, we also needed to confirm that these patients were sensitive to the allergens in question. For this purpose, we used skin prick tests (wheal ≥3 mm) or specific IgE measurements (≥0.35 kU/L). All these assessments were consistent with the diagnostic criteria published by the WAO in 2020 [12]. To include a case in the study, other possible triggers had to be ruled out, and the temporal relationship had to be clearly established.

The situation was somewhat more complex for idiopathic anaphylaxis. We classified cases of anaphylaxis that met the WAO/EAACI diagnostic criteria but for which we could not identify the triggering factor despite a comprehensive investigation into this category. This investigation included detailed history taking, skin prick tests, and specific IgE measurements for common allergens.

The severity of anaphylaxis cases is determined based on the clinical criteria defined in the World Allergy Organization (WAO) 2020 guide; it was evaluated in accordance with the European Academy of Allergy and Clinical Immunology (EAACI) 2021 guideline and the Turkish National Anaphylaxis Guideline (AID, 2018). Accordingly, only patients who clinically met the definition of anaphylaxis (patients with at least two system involvements or respiratory or circulatory system involvement alone) were included in the study. We excluded cases that only presented with skin or mucosal findings but no systemic involvement from the definition of anaphylaxis. In other words, even if a patient had local symptoms such as a skin rash or lip swelling, we did not classify the case as anaphylaxis if no systemic reaction developed.

The five-step classification of clinical severity defined in the WAO 2020 guideline [12] was simplified into three levels for practical application in our study:

- Mild reactions: Cases with mild respiratory or gastrointestinal involvement accompanying cutaneous findings and no life-threatening findings;
- Moderate reactions: Cases with significant respiratory or gastrointestinal involvement but no circulatory disorders;
- Severe reactions: Cases with respiratory failure, laryngospasm, hypotension, loss of consciousness or cardiovascular collapse.

This classification is used as a simplified version of the five-step system in the WAO 2020 guideline and is compatible with EAACI 2021 and the Turkish National Anaphylaxis Guideline 2018 [10,11,12].

### 2.2. Operating Procedures

The data collection process involved reviewing physical files through the hospital information management system. Using a standard data form, demographic information (age, gender, comorbidities), clinical findings and outcomes were systematically recorded. The clinical findings were organized into five sections: skin/mucosa, respiratory, gastrointestinal (GI), cardiovascular (CV), and neurological systems. Specific symptoms for each system (e.g., urticaria, pruritus, flushing; wheezing, stridor, laryngeal edema, hypotension, tachycardia, etc.) were individually marked and the number of organ systems involved was calculated.

The laboratory data included routine biochemical examination and blood gas results which clinicians used for validation and record verification but these results were excluded from quantitative analysis and reporting. Although tryptase measurement has been applied in the institution in recent years, it was not included in the analysis because it was not routinely performed in emergency room conditions and there were not enough records. The follow-up protocol required healthcare providers to document all observed events, including emergency department stay, biphasic reaction development, 72-h readmission, hospital admission requirements, hospital unit assignment, and duration of hospitalization. The development of a biphasic reaction occurred when patients experienced anaphylaxis symptoms again during the 72-h period following their initial symptom resolution. Information on mortality, prescribing epinephrine auto-injectors, patient or family education, and referral to an allergist was also added. All data were cross-confirmed by two researchers.

### 2.3. Treatment Protocol and Clinical Management

The current clinical methods received retrospective evaluation for their effectiveness without any intervention. The study documented epinephrine administration details through three categories, which included administration route (intramuscular, subcutaneous or intravenous), dose amount (mg/kg), the need for additional doses and the time interval from symptom onset to first epinephrine administration. The study documented all supportive treatments including corticosteroids, antihistamines, bronchodilators, intravenous fluids and oxygen therapy, as well as all cases needing critical care intervention, including epinephrine infusion and cardiopulmonary resuscitation (CPR).

The study determined quality indicator metrics by evaluating intramuscular epinephrine administration rate, initial thirty-minute epinephrine administration rates, proper dose administration, four-to-six-hour observation periods, auto-injector prescriptions, allergist referrals, patient and family education, and biphasic reaction monitoring rates. The research presented quantitative data about epinephrine dose amounts (mg/kg) and administration times through numerical values and supporting visual representations when needed.

In order to determine the cause of anaphylaxis, skin prick tests and/or specific IgE measurements were performed for suspected allergens based on history in patients with clinical indications. These tests were performed during the recovery period after an acute attack—usually at least 4–6 weeks later—in line with the WAO 2020 and EAACI 2021 guidelines. The evaluation of food and aeroallergen panels occurred only when doctors determined it was essential. Provocation tests were not performed in patients with a history of anaphylaxis for safety reasons; it was postponed to the future in high-risk cases. Patients whose triggers could not be determined despite the tests performed and who met the WAO/EAACI diagnostic criteria were classified as “idiopathic anaphylaxis” in accordance with the guidelines. The diagnosis of mast cell activation syndrome and other systemic causes, including urticaria, FPIES and hereditary angioedema was ruled out through both clinical evaluation and laboratory tests.

### 2.4. Statistical Analysis

Statistical analyses were performed using SPSS 26.0 software. The distribution of continuous variables was evaluated with the Shapiro–Wilk test, and the variance homogeneity was evaluated with the Levene test. Normally distributed variables were expressed as mean ± standard deviation (SD), and non-normally distributed variables were expressed as median (interquartile range, IQR). Categorical variables were presented as frequency (n) and percentage (%).

In the comparison of two independent groups, the Student’s *t*-test was used for normally distributed continuous variables and the Mann–Whitney U test was used for variables that were not normally distributed. In the analyses involving three or more groups, one-way ANOVA or Kruskal–Wallis test was applied depending on the distribution; when a significant difference was detected, a Bonferroni-corrected post-hoc test was used for the data with variance homogeneity, and Dunn’s test was used for non-parametric data. Chi-square test was used to compare categorical variables, but the Fisher exact test was preferred if the expected cell frequency was below 5.

Previously planned comparisons in the study included gender, trigger distribution, epinephrine administration rate, anaphylaxis severity, biphasic reaction, and hospitalization rates by age groups; the Chi-square or Fisher’s exact test was used in these analyses. Patients who were administered epinephrine and those who were not administered epinephrine were compared with the Student’s *t*-test in terms of age, and with Chi-square or Fisher tests in terms of gender and triggering factors. Appropriate statistical methods (*t*-test, Fisher or Mann–Whitney U) were applied for organ system findings, number of organs involved, severity of anaphylaxis, and observation period. Epinephrine dose (mg/kg) and time from symptom onset to first epinephrine administration (minutes) were analyzed as continuous variables and the results are presented in tables or figures.

Given the very small sample size in the non-epinephrine group (n = 4), statistical comparisons between epinephrine-treated and non-treated patients were interpreted with caution, and inferential statistics were limited to descriptive purposes only. No definitive conclusions were drawn from these comparisons due to insufficient statistical power.

The trigger-based severity analysis used a hierarchical system to assign primary triggers where Food took precedence over Drug, Venom, Aeroallergen, Idiopathic, and Other/Unknown triggers.

All tests were done with two tails; *p* < 0.05 was considered statistically significant. Values of *p* ≥ 0.01 were written in two decimal places, and values of *p* < 0.01 were written in three decimal places; *p* < 0.001 results were reported as “*p* < 0.001”. Cases with missing data were excluded from the analysis, and data imputation was not performed. Subgroup analyses were conducted taking into account age groups, trigger categories, epinephrine administration, and anaphylaxis severity as defined at baseline.

### 2.5. Units of Measurement

All measurements were reported on the basis of the International System of Units (SI): age (years), observation period and time from symptom to treatment (hours/minute), length of hospital stay (days), epinephrine dose (mg/kg), temperature (°C), and blood pressure (mmHg).

## 3. Results

### 3.1. Demographic Characteristics and Co-Morbidities

A total of 274 patients were found in the system in the retrospective screening with the diagnosis of anaphylaxis. After excluding patients who did not meet the diagnostic criteria for anaphylaxis and did not meet the inclusion criteria of the study, data from 166 patients were included in the analysis. The mean age was calculated as 7.4 years. When the distribution by age groups was examined, it was seen that the highest number of cases was found in the 0–2 age group (n = 49). This group was followed by 12–18 (n = 43), 6–11 (n = 43) and 3–5 (n = 31) age ranges, respectively. There was a slight superiority of males in the gender distribution (81 males). When evaluated in terms of concomitant allergic diseases, 62 cases (37.3%) had at least one comorbid condition. Asthma stood out as the most common comorbidity in 22 patients (13.3%). It was followed by atopic dermatitis (n = 7), allergic rhinitis (n = 3), and other conditions (n = 30), respectively (Table 1).

### 3.2. Anaphylaxis Triggering Factors

When the triggers causing anaphylaxis attacks were evaluated, it was determined that the most common factor was food (n = 88). There was a statistically significant difference in trigger distributions between age groups (*p* < 0.001). While food allergy was significantly dominant in younger age groups, the rate of drug-induced anaphylaxis increased with increasing age. Among food allergens, cow’s milk (n = 40), eggs (n = 26), and nuts/peanuts (n = 10) were the most common causes. A significant increase in drug-induced anaphylaxis cases was observed with age (n = 16); in the adolescent group, this was second only to foods. Penicillin and cephalosporin group antibiotics (n = 18) accounted for more than half of drug allergies. NSAIDs (n = 8) were a particularly notable cause in adolescence. Bee stings (n = 8) and aeroallergen-related cases (n = 18) were observed with lower frequency (Table 2).

### 3.3. Clinical Findings and Organ Involvement

When organ system involvement patterns were examined in anaphylaxis cases, skin and mucosa involvement were present in almost all patients (n = 155). Urticaria (n = 63) was the most common skin finding, while angioedema (n = 39) and flushing (n = 22) were also common symptoms. Respiratory system involvement was found to be high (n = 112); wheezing and bronchospasm (n = 50) were the most common respiratory findings. Thirty-two patients had shortness of breath, and five had life-threatening upper respiratory tract involvement such as laryngeal edema.

Gastrointestinal tract manifestations were observed in 56 patients. Vomiting (n = 37) was the most common symptom, followed by nausea (n = 10) and abdominal pain (n = 9). Cardiovascular system involvement was recorded in 27 patients, and hypotension (n = 25) was the most prominent finding. Shock did not develop in any patient. Neurological system involvement was the least common organ involvement at a rate of 2.4% (n = 4). In general, two or more organ systems were affected in most cases. The mean number of organ systems involved was found to be 2.2. The severity of anaphylaxis was assessed in 164 patients according to the Sampson criteria. As a result of the evaluation, mild (n = 16), moderate (n = 62) and severe (n = 86) anaphylaxis cases were detected. Since the data of two patients were incomplete, severity could not be evaluated (Table 3).

Among the 166 patients, 11 (6.6%) presented with anaphylaxis without any skin or mucosal manifestations. The subgroup presented respiratory symptoms which became their primary clinical manifestation and affected nine patients (81.8%) and cardiovascular symptoms affected five patients (45.5%). The time period from patient arrival to epinephrine administration took 45 min for patients without skin symptoms, yet their IQR ranged from 25 to 70 min. The time to epinephrine administration for patients showing skin symptoms was 28 min with an IQR ranging from 15 to 50 min (*p* = 0.032). The delay in diagnosis might stem from physicians being unsure about the diagnosis due to the absence of skin symptoms. The severity distribution of patients showed that two patients (18.2%) had mild anaphylaxis, while three patients (27.3%) had moderate anaphylaxis and six patients (54.5%) had severe anaphylaxis.

The study results in Table 4 show that drug-induced anaphylaxis produced the most severe reactions at 81.6%, followed by venom anaphylaxis at 75.0% and food anaphylaxis at 46.6%. Research shows that drug-induced anaphylaxis results in elevated death rates according to studies [4,9]. Notably, none of the aeroallergen-related cases were classified as severe; all seven cases presented with moderate severity. The food-induced anaphylaxis study shows that 46.6% of patients developed severe reactions which exceeds the typical Western population rates of 30–40%. The study results might show different clinical classification methods and patients may have delayed their presentation to healthcare facilities. The data distribution showed that trigger types produced significant statistical effects (*p* < 0.001) which established their corresponding severity levels.

### 3.4. Treatment Approaches and Interventions

The emergency department staff used epinephrine as a treatment for 162 patients (97.6%) during their assessment. Although anaphylaxis was diagnosed, only four patients (2.4%) were not given epinephrine; all of these cases were classified as mild anaphylaxis (n = 4). In addition to epinephrine, corticosteroids were used in 156 patients (94.0%) and H1 antihistamines were used in 152 patients (91.6%). H2 blocker treatment was applied in 12 patients (7.2%). The treatment included inhaled bronchodilators for 64 patients, intravenous fluid support for 42 patients and oxygen therapy for 38 patients who needed it clinically.

**Figure 1:**
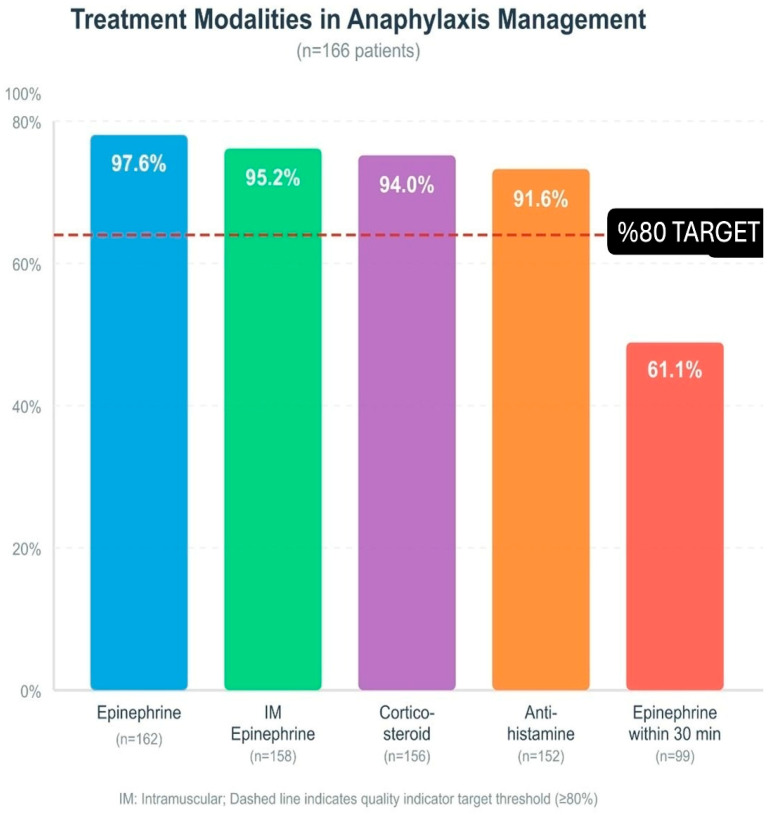
Treatment modalities in anaphylaxis management (n = 166): epinephrine, IM epinephrine, corticosteroid, antihistamine, epinephrine within 30 min. Dashed line: ≥80% target; IM: intramuscular.

When the route of epinephrine administration was evaluated, intramuscular injection was by far the most preferred method in 158 cases. A small number of patients were treated by the intravenous route (four patients). In some cases, a single dose of epinephrine was not sufficient; eight patients (4.8%) required a second dose. The medical team administered epinephrine infusion to four patients (2.4%) and performed cardiopulmonary resuscitation (CPR) on two patients (1.2%). The calculated median epinephrine dose of 0.01 mg/kg matched the recommended amounts in current medical guidelines. The first epinephrine administration occurred at a median time of 30 min after symptom appearance (Figure 1).

### 3.5. Comparative Analysis of Adrenaline Use

When comparing 162 patients who received epinephrine and 4 patients who did not, there were some remarkable differences, although the group that did not receive epinephrine was quite small. Given the very small sample size in the non-epinephrine group (n = 4), statistical comparisons were interpreted with caution and limited to descriptive purposes only. No significant difference was observed between the groups in terms of age (7.6 ± 5.6 vs. 5.0 ± 4.0 years, *p* = 0.137), gender (48.1% vs. 50.0% male, *p* = 0.921), and triggering factors (food: 52.5% vs. 50.0%). In both groups, skin findings were present in almost all patients (150 vs. 4). Respiratory system symptoms were more common in the epinephrine group, with 111 versus 2 cases and cardiovascular involvement with 26 versus 1 cases, but this difference was not statistically significant. There was also no significant difference between gastrointestinal findings (56 vs. 1) and the mean number of organ systems involved (2.2 vs. 2.0).

**Figure 2:**
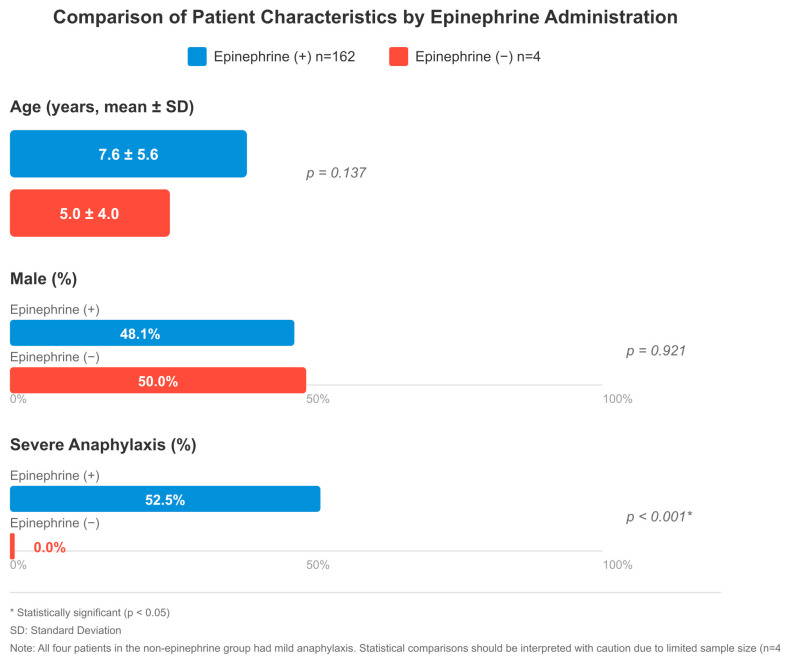
Comparison of patient characteristics by epinephrine administration: age (mean ± SD), male (%), and severe anaphylaxis (%). The epinephrine-treated group included 162 patients; severity was assessable in 160, of whom 85 (53.1%) had severe anaphylaxis. Two patients had incomplete severity data. All four patients in the non-epinephrine group had mild anaphylaxis; no severe cases were present in this group. * *p* < 0.05 indicates statistical significance. SD: standard deviation. Note: Statistical comparisons involving the non-epinephrine group (n = 4) should be interpreted with caution due to limited sample size.

When the severity of anaphylaxis was evaluated, a significant difference was found between the groups (*p* < 0.001). All four patients who were not administered epinephrine had mild anaphylaxis. In the epinephrine-treated group, mild anaphylaxis was observed in 12 patients (7.4%), moderate in 62 (38.3%), and severe in 85 (52.5%). The 85 patients who had severe anaphylaxis received epinephrine as their treatment but the non-epinephrine group did not have any severe cases. In terms of observation period, patients who were given epinephrine were followed for a longer period of time (*p* = 0.184). There was no significant difference in hospitalization rates (*p* = 0.713) (Figure 2).

### 3.6. Follow-Up Period and Clinical Outcomes of the Patients

When the observation period in the emergency department was examined, it was seen that the median duration was 6 h. In total, 156 of the patients were followed for at least 4 h in accordance with the guidelines. Biphasic reaction developed in 10 patients (6.0%); 2 patients (1.2%) came back to the emergency department within 72 h of the first admission. The hospitalization rate was 4.8%. Two of the inpatients were admitted to the intensive care unit and six to the ward. The remaining 156 patients were discharged after the recommended observation period of at least 4 h. The median length of hospital stay for inpatients was 2 days, and no deaths were found in the study. In the post-discharge period, 13 patients were prescribed epinephrine auto-injectors. In addition, 140 patients and their families were trained, and 26 patients were referred to an allergist for further evaluation (Table 1).

### 3.7. Comparison of Clinical Features by Age Groups

The study showed that gender distribution between different age groups differed substantially (*p* = 0.003). There were significant and statistically significant differences in the distribution of triggering factors by age (*p* < 0.001). The youngest age group showed the highest rate of food allergy at 85.7%, but drug-induced cases became more common as age increased, up 37.2% in adolescents. The number of bee sting-related cases together with other causes, demonstrated an age-related increase. The rate of epinephrine administration was high and close to each other in all age groups (range 95–98%). There was a statistically significant difference between the groups in terms of anaphylaxis severity (*p* = 0.042); the rate of severe cases increased with age, from 40.8% in the 0–2 age group to 62.8% in the 12–18 age group. The distribution of biphasic reaction development and hospitalization rates followed the same pattern across different age groups (Table 2 and Table 3).

### 3.8. Quality Indicators and Clinical Compliance Rates

**Figure 3:**
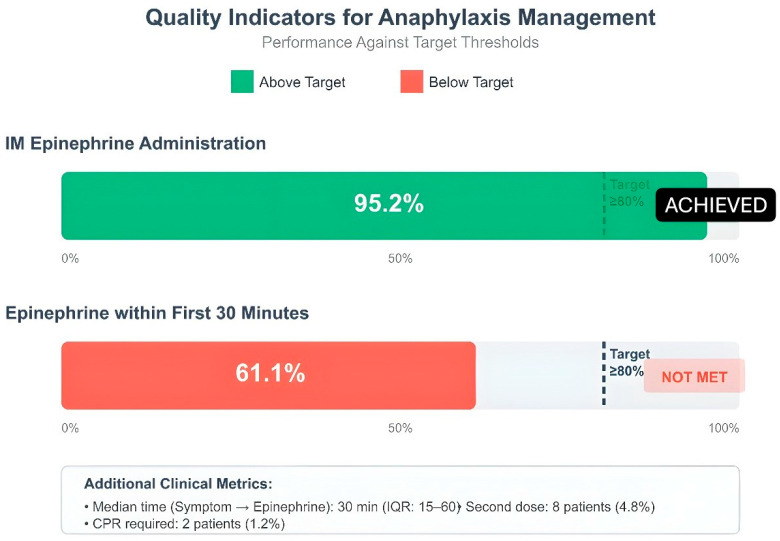
Quality indicators for anaphylaxis management: IM epinephrine administration, epinephrine within first 30 min. Dashed line: ≥80% target; green: achieved, red: not met; IM: intramuscular; IQR: interquartile range.

The evaluation of pediatric anaphylaxis management quality indicators revealed that healthcare providers followed guidelines well in certain aspects yet needed to enhance their performance in specific areas. Intramuscular epinephrine administration achieved very high adherence rates in 158 patients (95.2%) in terms of correct dose (0.01 mg/kg) use, which was above the target threshold (80%). The first 30 min of treatment included epinephrine administration for 99 patients (61.1%). The 6-h observation period included 156 patients who successfully completed the 4-h follow-up requirement (93.9%). The rate of referral to an allergist was 15.7% (n = 26). The rate of patient and family education reached a high value of 84.3%. Monitoring for biphasic reaction was performed in all 166 patients and full compliance was achieved in this indicator. The prescription rate for epinephrine auto-injectors stayed at 7.8%, which fell short of the desired 80% target threshold. The team recognized this area required enhancement because it directly affected patient safety following hospital discharge (Figure 3).

## 4. Discussion

The research investigated how anaphylaxis presents in children and what emergency department protocols exist for their immediate medical needs. The research findings show that various age groups have different triggers that activate their responses. The research indicated that food allergies triggered most allergic reactions in children but drug allergies started to appear more frequently during their transition to adulthood. The rate of intramuscular epinephrine use in our center was quite high and it was observed that early intervention protocols were applied effectively. In general, it can be said that the recommendations in international guidelines are largely followed, but there are some areas of improvement in the post-discharge processes.

Several aspects of this study contribute novel insights to the literature on pediatric anaphylaxis management. The study conducts a complete evaluation of pediatric anaphylaxis quality indicators that operate at a Turkish tertiary medical facility to fill an essential knowledge void in the region. The research shows that patients demonstrate excellent acute-phase treatment compliance through their receipt of IM epinephrine at 97.6% and their exact adherence to prescribed dosages at 100% yet they do not receive appropriate follow-up care after discharge because only 7.8% of patients receive auto-injector prescriptions. The study reveals an unreported connection between this patient group which requires healthcare organizations to develop discharge protocols as their main quality improvement focus. Third, our study which analyzed patients by age group showed that our population starts experiencing drug-induced anaphylaxis rather than food-induced anaphylaxis at a younger age than some Western populations might experience.

The main factor that triggered the cohort was food allergy, because it occurred at a rate of 53%. This rate seems to be quite consistent with the 50–70% range reported in the literature [13,14]. The research results demonstrated that food allergies appeared in children under two years old at a rate of 85.7% which confirmed earlier studies [13,14]. The study showed that cow’s milk caused 24.1% of reactions and eggs triggered 15.7% of the allergic reactions. These rates coincide with reported values in the Asian and European cohorts (cow’s milk 15–35%, eggs 15–25%) [13,14]. The study shows that cow’s milk causes anaphylaxis at a rate of 51.0% in infants between 0 and 2 years, which surpasses what has been documented in certain Western studies. The high rate of cow’s milk-induced anaphylaxis in infants might result from our population’s practice of giving cow’s milk formula at an early age and following specific dietary habits. The immaturity of the intestinal barrier in early childhood and the development of immune tolerance are seen as a possible mechanism explaining the strong allergenic effect of these proteins. Nuts came to the fore with a rate of 12%, especially among school-age and adolescents. The research data shows that sensitivity levels increase with age according to two independent studies [13,14]. The research showed that drug-induced anaphylaxis appeared infrequently in infants but it became the most common cause of anaphylaxis, at 37.2%, in people between 12 and 18 years old (*p* < 0.001). This increase may be related to the increase in antibiotic and analgesic use during adolescence [15,16]. Beta-lactams emerged as the leading drug trigger because they accounted for 10.8% of all reactions. Research from around the world shows penicillin and cephalosporins frequently trigger allergic reactions [15,16]. The 4.8% of patients who reacted to NSAIDs showed a high incidence among adolescent patients. The Turkish tertiary center reported that medications started causing most allergic reactions which affected 54% of patients, while cow’s milk proved to be the main food allergen that affected 33.3% of infants with food allergies [3]. The immune system’s development through childhood to adolescence explains why food allergies occur most in young children but drug allergies become more common during adolescence.

The research team gave epinephrine to 97.6% of all patients who received treatment. This is significantly higher than the 34.1% reported by Lin et al. [17]. It is known in the literature that epinephrine use in emergency departments is generally below 50% [18]. The current data from tertiary health facilities show that epinephrine administration succeeds in 94.8–100% of cases when medical staff receive proper training and when patients visit pediatric allergy clinics [3]. The research data (97.6%) confirms this observation because institutions that train their staff and maintain dedicated specialist teams will achieve better results in guideline adherence. The different results between our center and other hospitals demonstrate how well our facility follows established protocols. The intramuscular route was preferred in 95.2% of the patients who received epinephrine, and this approach was in full compliance with international guidelines [18]. The fast absorption of intramuscular administration occurs through this method, but intravenous bolus delivery poses dangers that include heart rhythm disturbances [18]. Research studies have proven that intramuscular epinephrine injections deliver the same level of resuscitation success as intravenous administration [19]. The study results showed that healthcare providers administered epinephrine at a rate of 61.1% during the initial thirty minutes. The researchers measured the time from symptom appearance to epinephrine treatment at 30 min with a range of 15 to 60 min. Medical research shows that giving epinephrine treatment at the start prevents biphasic reactions from occurring [20]. Research shows that patients treated with epinephrine outside hospitals experienced lower rates of biphasic reactions (5.4% vs. 9.3%) [21]. Healthcare providers choose to delay epinephrine administration because they either doubt the diagnosis or want to monitor patients who display only mild symptoms [17]. The majority of anaphylaxis-related deaths happen during the first sixty minutes so healthcare providers must start treatment right away [17]. The researchers determined that the average epinephrine dosage reached 0.01 mg/kg, which followed the recommended guidelines [18]. The requirement for a second dose of epinephrine occurred in 4.8% of patients. Research shows that 90% of anaphylaxis cases need only one dose of treatment but 10% require multiple doses of medication [18]. European medical records show that 2.3% of patients required an additional epinephrine dose [18]. The requirement for additional epinephrine doses typically indicates that patients received their diagnosis too late or their heart condition was severe or they took beta-blockers [18]. The researchers performed epinephrine infusion on 2.4% of patients and performed CPR on 1.2% of patients. Research studies show that refractory anaphylaxis develops in 0.36% to 2.3% of all anaphylaxis cases [18]. The death rate from anaphylaxis that occurs during surgical procedures amounts to 1.4% to 4.8% but anaphylaxis cases found in the general public typically result in less than 1% mortality [18]. All four patients who were not administered epinephrine had mild anaphylaxis (*p* < 0.001); no severe cases were present in the non-epinephrine group (Figure 2). Given the very small sample size (n = 4), these comparisons were interpreted with caution and limited to descriptive purposes only. The study by Lin et al. demonstrated that antihistamine and corticosteroid treatment should not be used as the primary treatment for these cases [17]. However, the risk of death continues in these patients; most of the deaths are seen within the first hour [17]. Dribin and Greenhawt demonstrated that immediate epinephrine treatment helps prevent biphasic reaction development according to their study [20]. Most of the hesitations that cause delay are due to fear of side effects [17].

In our study, skin and mucosal membrane involvement was seen in 93.4% of the patients. The most common finding was urticaria (38.0%), followed by angioedema (23.5%). The research results from this study confirmed the results that Caliskan et al. had previously discovered [3]. Respiratory system involvement ranked second with a rate of 67.5%. Wheezing and bronchospasm stood out as the most common respiratory findings with a rate of 30.1%. Laryngeal edema was seen in 3.0% of patients and was significantly associated with a severe course [21]. Gastrointestinal involvement was observed in 33.7% of patients; vomiting is the most common symptom in this group with 22.3%. Cardiovascular involvement was 16.3%; hypotension was detected in 15.1%, and no shock was observed. These parameters are considered important predictors for severe anaphylaxis [21]. Neurological involvement was observed at the lowest rate with 2.4%. The mean number of organs involved was calculated as 2.2 ± 0.4. It is known that the need for intensive care increases in patients with multiple organ involvement [21]. The rate of severe anaphylaxis was found to be 52.4%. Shehataa and Salama reported that chest tightness, hypotension, hypoxia and tachypnea were early severe predictors [22]. Gani et al. stated that intraoperative hypotension and bronchospasm predict poor prognosis, and that an end-tidal CO_2_ value below 25 mmHg shows sensitivity and specificity of over 90% [21]. Concomitant asthma was present in 13.3% of the patients, and atopic dermatitis was present in 4.2% of the patients. It has been reported in the literature that poorly controlled asthma is associated with severe anaphylaxis, and atopic phenotypes increase the risk of anaphylaxis [23]. Knyziak-Mędrzycka et al. described that early food allergy and atopic dermatitis progress to asthma through the “atopic march” process [23]. It has been emphasized that personalized immunotherapy can be planned in these patients thanks to molecular diagnostic methods [24].

Among the 166 patients in our study, 11 (6.6%) presented with anaphylaxis without any skin or mucosal manifestations. In this subgroup, respiratory symptoms were the predominant finding (81.8%), followed by cardiovascular involvement (45.5%). The time it took for physicians to administer epinephrine to patients without skin symptoms reached 45 min, but patients who showed skin symptoms received epinephrine within 28 min (*p* = 0.032). The extended time period might indicate that medical staff struggled to identify anaphylaxis because they lacked visible skin symptoms, which demonstrates the need to identify unusual anaphylaxis symptoms.

The study results in Table 4 show that drug-induced anaphylaxis produced the most severe reactions at 81.6% followed by venom anaphylaxis at 75.0% and food anaphylaxis at 46.6%. Research shows that drug-induced anaphylaxis results in elevated death rates according to studies [4,9]. Notably, none of the aeroallergen-related cases were classified as severe; all seven cases presented with moderate severity. The study shows that food-induced anaphylaxis cases with severe symptoms make up 46.6% of total cases which exceeds the typical Western population rates of 30–40%. The study shows higher severe case rates than Western studies because patients might seek medical help later or physicians use different diagnosis methods or populations in this region consume different foods. The data distribution showed that trigger types establish strong statistical connections with their corresponding severity levels at a a *p*-value that is less than 0.001. The study found that age groups showed a statistically significant difference regarding their anaphylaxis severity levels (*p* = 0.042). The rate of severe anaphylaxis cases rose with age, starting from 40.8% in the 0–2 age group until it reached 62.8% in the 12–18 age group which might be because drug-induced anaphylaxis occurs more frequently among teenagers.

The biphasic reaction rate was found to be 6.0%. Gani et al. reported that the negative predictive value was 95% at one-hour follow-up and 97.3% at six-hour follow-up [21]. The proportion of patients hospitalized in intensive care was 1.2%, the proportion of patients hospitalized in the ward was 3.6%, and the total hospitalization rate was calculated as 4.8%. The study by Caliskan et al. [3] found that 3% of their pediatric emergency patients required PICU hospitalization. The median hospital stay was two days and no mortality cases were encountered. Shehataa and Salama reported that early diagnosis and timely treatment largely prevented mortality [22]. The median observation period was six hours, and 93.9% of the patients were monitored for at least four hours. Gaffney et al. reported that the majority of biphasic reactions developed within the first four hours, while late-onset cases were usually mild [25]. Current guidelines also recommend at least four hours of observation [25]. It is recommended to extend the follow-up period in patients with hypotension, persistent wheezing, multiple system involvement or the need for three doses of epinephrine [25]. In our study, the biphasic reaction developed at a rate of 6.0%; this value is consistent with the 5% rate reported in the literature [25]. All biphasic reactions were detected within four to six hours. Early epinephrine administration has also been shown to reduce this risk in various studies [26]. Complete monitoring was achieved in all patients (100%).

The rate of epinephrine auto-injector prescribing was well below the target at 7.8%. It is reported in the literature that this rate is around 60% [26]. Caliskan et al. reported an auto-injector prescription rate of 75.3% at discharge in their Turkish cohort [3]. The two Turkish tertiary centers showed a major difference between their post-discharge practices because they reported 7.8% versus 75.3% of patients received follow-up care after discharge. Urbani et al. demonstrated that doctors wrote fewer prescriptions because they underestimated the severity of the condition and lost contact with patients. Ziyar et al. demonstrated that auto-injector usage remained low because of both financial costs and psychological obstacles [27,28]. The EAACI guidelines establish auto-injector prescriptions as the recommended treatment for patients who experience severe anaphylaxis triggered by food or exercise [27]. Research shows that educational programs combined with visual materials and electronic reminder systems lead to substantial increases in prescription numbers [26,28]. The study by Ziyar et al. showed that patients who received interventions demonstrated a 38% increase in always carrying their auto-injectors and achieved a 70% improvement in correct usage [28]. The study by Urbani et al. demonstrated that patients who received continuous care from their doctors experienced a fivefold decrease in lost prescriptions (*p* < 0.0005), yet their actual medication use remained at 1.7% [27].

The patient education rate reached 84.3%. The main obstacles to maintaining long-term educational programs stem from healthcare staff departures and changes in their professional conduct according to Agbim et al. [26]. The training program should teach patients to stay away from triggers, identify early signs of anaphylaxis, demonstrate auto-injector usage and schedule regular medical check-ups [26]. The study found that 15.7% of patients received allergist referrals, which fell short of the 50% target when organizations implement effective quality improvement strategies. The follow-up appointment attendance rates for Medicaid patients reached the 17% mark documented in previous research [26]. Research indicates that the referral rate can reach 82%, while privately insured patients attended 43% of their scheduled appointments [26]. The re-application rate was discovered to be 1.2%. Gaffney et al. achieved a 1.6 percentage point decrease in re-application rates through their identical intervention approach [25]. Agbim et al. identified three main factors that led to re-applications: patients received insufficient training and experienced biphasic reactions and struggled to obtain auto-injectors [26]. The quality indicators in our study showed excellent results because intramuscular epinephrine administration reached 95.2%, dose accuracy was 100% and epinephrine delivery within 30 min occurred at 61.1% rate. The hospitalization rate for all patients reached 4.8%. The hospitalization rate decreased from 28.5% to 11.2% according to Gaffney et al. after they implemented their series of interventions [25]. The study reported no deaths during the observation period. The implementation of electronic order-sets together with scheduled clinician training sessions and scheduled quality assessment systems will help improve process performance.

The retrospective design of the study led to some limitations. The collection of data in laboratory settings becomes difficult because there exists no standardized approach to measure tryptase biomarkers. In addition, some clinical details could not be fully accessed due to retrospective data recording. The research analysis power became restricted because the study contained only four participants in the non-epinephrine group. The results from these analyses need special interpretation because their statistical power remains limited. However, despite the fact that the study was single-center, it included a large patient group, a long observation period, and a detailed clinical evaluation protocol, which stand out as important strengths.

Future studies need to evaluate prehospital treatment effectiveness through molecular diagnostic tests that track patient recovery from the time of hospital discharge. The evaluation of anaphylaxis management programs in real-world clinical settings will result in increased auto-injector prescriptions and enhanced clinical practices. The development of national benchmarks for pediatric anaphylaxis management needs research from multiple centers to evaluate quality indicators between Turkish centers for determining their best practices.

## 5. Conclusions

The research investigates pediatric anaphylaxis treatment at a Turkish tertiary medical center to obtain vital information. The research found different age-specific patterns that cause anaphylaxis in children during this study. Foods in young children and drugs in older age groups have come to the fore as the main cause. Our center achieved excellent compliance with international acute-phase treatment guidelines through proper intramuscular epinephrine therapy administration at high rates, but we discovered that patient readiness following hospital discharge did not match the emergency care standards we achieved. The auto-injector prescription rate and allergist referral rate show significant differences from international standards, which need quality improvement interventions to address these issues. Another noteworthy finding was that the group with the highest incidence of severe cases was drug-induced anaphylaxis. We also observed significant delays in administering adrenaline to patients without skin manifestations. This situation once again highlights how critical it is to recognise atypical presentations.

Standardized discharge protocols need to exist for the proper management of childhood anaphylaxis cases. The protocols need to contain two vital elements, which involve providing auto-injectors to patients at risk and creating a uniform process for allergy specialist referrals. The upcoming research phase will evaluate the increased impact of these interventions through studies that will take place at various research facilities. The research needs to focus on two critical domains, which involve studying why anaphylaxis happens multiple times and developing methods for patients to manage their condition outside medical facilities.

## Figures and Tables

**Table 1:** Demographic Characteristics, Comorbidity and Outcomes.

Parameters	n (%)
Total patients	166 (100)
Age groups	
0–2 years	49 (29.5)
3–5 years	31 (18.7)
6–11 years	43 (25.9)
12–18 years	43 (25.9)
Gender (Male)	81 (48.8)
Comorbidity	62 (37.3)
- Asthma	22 (13.3)
- Atopic dermatitis	7 (4.2)
- Allergic rhinitis	3 (1.8)
- Other	30 (18.1)
Biphasic reaction	10 (6.0)
Hospitalization	8 (4.8)
Discharged	156 (93.9)
Mortality	0 (0.0)
Auto-injector prescription	13 (7.8)
First attack	140 (84.3)

Male gender distribution showed a statistically significant increase with age (*p* = 0.003). AD, atopic dermatitis; AR, allergic rhinitis.

**Table 2:** Etiology of Anaphylaxis, Triggering Factors and Distribution by Age Groups.

Triggering Factor	Total n (%)	0–2 Years n (%)	3–5 Years n (%)	6–11 Years n (%)	12–18 Years n (%)
Food	88 (53.0)	42 (85.7)	18 (58.1)	16 (37.2)	12 (27.9)
- Cow’s milk	40 (24.1)	25 (51.0)	7 (22.6)	8 (18.6)	0 (0.0)
- Egg	26 (15.7)	20 (40.8)	6 (19.4)	0 (0.0)	0 (0.0)
- Hazelnut/peanut	10 (6.0)	4 (8.2)	1 (3.2)	4 (9.3)	1 (2.3)
- Other nuts	10 (6.0)	4 (8.2)	3 (9.7)	2 (4.7)	1 (2.3)
- Pulses	2 (1.2)	0 (0.0)	1 (3.2)	0 (0.0)	1 (2.3)
Drug	41 (24.7)	4 (8.2)	5 (16.1)	16 (37.2)	16 (37.2)
- Penicillin/cephalosporin	18 (10.8)	4 (8.2)	2 (6.5)	9 (20.9)	3 (7.0)
- NSAIDs	8 (4.8)	0 (0.0)	0 (0.0)	2 (4.7)	6 (14.0)
- Other (IVIG, etc.)	15 (9.0)	0 (0.0)	3 (9.7)	5 (11.6)	7 (16.3)
Venom	8 (4.8)	0 (0.0)	1 (3.2)	3 (7.0)	4 (9.3)
Aeroallergen †	18 (10.8)	2 (4.1)	6 (19.4)	6 (14.0)	4 (9.3)
Other/unknown	25 (15.1)	3 (6.1)	5 (16.1)	7 (16.3)	10 (23.3)

Total patients: 166. Age groups: 0–2 years (n = 49), 3–5 years (n = 31), 6–11 years (n = 43), 12–18 years (n = 43). Total triggers: 180 (8.4% had multiple triggers). Statistics: Chi-square test; *p* < 0.05. † Aeroallergen-related anaphylaxis was defined as systemic anaphylactic reactions occurring during or within 30 min of documented exposure to airborne allergens (e.g., pollen, mold spores, animal dander) in patients with confirmed sensitization via skin prick testing (wheal ≥ 3 mm) and/or specific IgE measurement (≥0.35 kU/L), consistent with WAO 2020 diagnostic criteria.

**Table 3:** Clinical Findings, Organ Involvement, Severity and Distribution by Age Groups.

Parameters	Total n (%)	0–2 Years n (%)	3–5 Years n (%)	6–11 Years n (%)	12–18 Years n (%)	*p*-Value
Skin/mucosa	155 (93.4)	46 (93.9)	29 (93.5)	41 (95.3)	39 (90.7)	0.876
- Urticaria	63 (38.0)	–	–	–	–	
- Angioedema	39 (23.5)	–	–	–	–	
Respiratory	112 (67.5)	28 (57.1)	20 (64.5)	31 (72.1)	33 (76.7)	0.134
Gastrointestinal	56 (33.7)	22 (44.9)	11 (35.5)	12 (27.9)	11 (25.6)	0.156
Cardiovascular	27 (16.3)	5 (10.2)	4 (12.9)	8 (18.6)	10 (23.3)	0.298
Neurological	4 (2.4)	1 (2.0)	1 (3.2)	1 (2.3)	1 (2.3)	0.993
Number of organ systems involved (mean ± SD)	2.2 ± 0.4	2.1 ± 0.6	2.2 ± 0.5	2.3 ± 0.5	2.3 ± 0.5	0.112
Severity †						0.042 *
- Mild	16 (9.8)	8 (16.3)	4 (12.9)	1 (2.3)	3 (7.0)	
- Moderate	62 (37.8)	21 (42.9)	12 (38.7)	17 (39.5)	12 (27.9)	
- Severe	86 (52.4)	20 (40.8)	15 (48.4)	24 (55.8)	27 (62.8)	

Statistics: Chi-square/Fisher’s exact test; * *p* < 0.05. † Severity was assessed in 164 patients according to Sampson criteria; data were incomplete for 2 patients. SD, standard deviation.

**Table 4:** Severity Distribution of Anaphylaxis Stratified by Trigger Type.

Trigger	Mild n (%)	Moderate n (%)	Severe n (%)	Total
Food	12 (13.6)	35 (39.8)	41 (46.6)	88
Drug	0 (0.0)	7 (18.4)	31 (81.6)	38
Venom	0 (0.0)	2 (25.0)	6 (75.0)	8
Aeroallergen	0 (0.0)	7 (100.0)	0 (0.0)	7
Idiopathic	2 (12.5)	8 (50.0)	6 (37.5)	16
Other/Unknown	2 (22.2)	3 (33.3)	4 (44.4)	9
Total	16 (9.6)	62 (37.3)	88 (53.0)	166

Severity was assessed according to Sampson criteria. Primary trigger was assigned hierarchically (Food > Drug > Venom > Aeroallergen > Idiopathic > Other/Unknown) for patients with multiple potential triggers; therefore, trigger-specific totals may differ from Table 2. Severity data were incomplete for 2 patients (as noted in Table 3); the total severe count (n = 88) reflects trigger-based classification where severity could be determined from clinical records for these cases. Statistics: Chi-square test.

## Data Availability

The data presented in this study are available on request from the corresponding author. The data are not publicly available due to ethical and privacy restrictions.

## Abbreviations

AAAAI	American Academy of Allergy, Asthma & Immunology
CPR	Cardiopulmonary Resuscitation
CVS	Cardiovascular System
EAACI	European Academy of Allergy and Clinical Immunology
GIS	Gastrointestinal System
IM	Intramuscular
IQR	Interquartile Range
MRGPRX2	Mas-Related G Protein–Coupled Receptor X2
NSAID	Nonsteroidal Anti-Inflammatory Drug
SD	Standard Deviation
SI	International System of Units
SPSS	Statistical Package for the Social Sciences
WAO	World Allergy Organization
